# *Metschnikowia pulcherrima* Influences the Expression of Genes Involved in PDH Bypass and Glyceropyruvic Fermentation in *Saccharomyces cerevisiae*

**DOI:** 10.3389/fmicb.2017.01137

**Published:** 2017-06-28

**Authors:** Mohand Sadoudi, Sandrine Rousseaux, Vanessa David, Hervé Alexandre, Raphaëlle Tourdot-Maréchal

**Affiliations:** UMR Procédés Alimentaires Microbiologiques - Université de Bourgogne Franche-Comté/AgroSup Dijon - équipe Vin ALiments Micro-organismes Stress, Institut Universitaire de la Vigne et du Vin Jules Guyot, Université de BourgogneDijon, France

**Keywords:** sequential culture *Metschnikowia pulcherrima/Saccharomyces cerevisiae*, acetic acid, glycerol, alcoholic fermentation, quantitative RT-PCR

## Abstract

Previous studies reported that the use of *Metschnikowia pulcherrima* in sequential culture fermentation with *Saccharomyces cerevisiae* mainly induced a reduction of volatile acidity in wine. The impact of the presence of this yeast on the metabolic pathway involved in pyruvate dehydrogenase (PDH) bypass and glycerol production in *S. cerevisiae* has never been investigated. In this work, we compared acetic acid and glycerol production kinetics between pure *S. cerevisiae* culture and its sequential culture with *M. pulcherrima* during alcoholic fermentation. In parallel, the expression levels of the principal genes involved in PDH bypass and glyceropyruvic fermentation in *S. cerevisiae* were investigated. A sequential culture of *M. pulcherrima*/*S. cerevisiae* at an inoculation ratio of 10:1 produced 40% less acetic acid than pure *S. cerevisiae* culture and led to the enhancement of glycerol content (12% higher). High expression levels of pyruvate decarboxylase *PDC1* and *PDC5*, acetaldehyde dehydrogenase *ALD6*, alcohol dehydrogenase *ADH1* and glycerol-3-phosphate dehydrogenase *PDC1* genes during the first 3 days of fermentation in sequential culture conditions are highlighted. Despite the complexity of correlating gene expression levels to acetic acid formation kinetics, we demonstrate that the acetic acid production pathway is altered by sequential culture conditions. Moreover, we show for the first time that the entire acetic acid and glycerol metabolic pathway can be modulated in *S. cerevisiae* by the presence of *M. pulcherrima* at the beginning of fermentation.

## Introduction

Complex interactions between organisms occur when fermentations are conducted with different yeasts (Fleet, 2003; Alexandre et al., 2004; Liu et al., 2015; Albergaria and Arneborg, 2016; Ciani et al., 2016). Considerable differences have been shown in the metabolism of *Saccharomyces cerevisiae* in single and in co-culture with non-*Saccharomyces* yeasts. Moreira et al. (2005) reported an increase in the quantity of desirable compounds, such as higher alcohols and esters, when *S. cerevisiae* was co-fermented with *Hanseniaspora uvarum*. A previous study (Sadoudi et al., 2012) based on the analysis of 48 volatile compounds belonging to different chemical families, highlighted the existence of different types of interactions independent of biomass production between non-*Saccharomyces* yeasts co-cultured with *S. cerevisiae*. More precisely, a positive interaction (synergistic effect) between *Metschnikowia pulcherrima* and *S. cerevisiae* resulted in a higher level of aromatic compounds than the sum of the aromatic compounds present in each monoculture. In addition, in a sequential *M. pulcherrima*/*S. cerevisiae* culture, acetic acid production was significantly lower compared to that obtained with a *S. cerevisiae* monoculture. Different studies reported low acetic acid production for certain non-*Saccharomyces* yeasts (*M. pulcherrima*, *Torulaspora delbrueckii*, *Starmerella bacillaris*) and their capacity in culture with *S. cerevisiae* to produce lower acetic acid concentrations in comparison to *S. cerevisiae* monoculture (Bely et al., 2008; Comitini et al., 2011; Milanovic et al., 2012; Rantsiou et al., 2012). These studies suggest that the acetic acid metabolic pathway can be affected by interactions occurring between yeasts, leading to a decrease in the amount of acetic acid. However, little is known as yet of the impact of sequential non-*Saccharomyces*/*S. cerevisiae* culture on the genes involved in the acetic acid metabolic pathway of *S. cerevisiae*.

Acetic acid is the principal volatile acid of wine. It has a negative impact on yeast fermentative performance and affects the quality of some wines when present above a given concentration (Rasmussen et al., 1995). The OIV (2010) states that the maximum acceptable limit for volatile acidity for most wines is 1.2 g l^-1^ of acetic acid. Unfortunately, higher levels are sometimes produced, depending on the strain (Erasmus et al., 2004; Orlić et al., 2010), on grape or must composition (Delfini and Costa, 1993) and on the winemaking process (Barbosa et al., 2009). Therefore, strains with reduced acetate production would have a high enological value. Studies on the production of volatile acidity by *S. cerevisiae* in winemaking conditions showed that this acid is mainly formed at the beginning of alcoholic fermentation (Alexandre et al., 2004; Bely et al., 2008). Acetic acid is formed rapidly during the fermentation of the first 50–100 g l^-1^ of sugar, but part of it is metabolized by *S. cerevisiae* (Ribéreau-Gayon et al., 2006). This yeast can also assimilate acetic acid added at the beginning of alcoholic fermentation (Vasserot et al., 2010).

**FIGURE 1:**
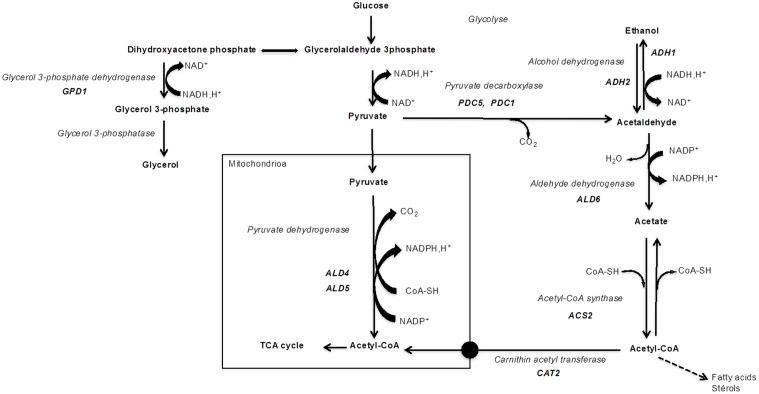
Enzymes and main genes involved in PDH bypass and in glycerol production (glyceropyruvic fermentation).

Acetic acid is a by-product of alcoholic fermentation produced *via* the pyruvate dehydrogenase (PDH) bypass (**Figure 1**). It is produced at the onset of anaerobic growth conditions, as a reducing equivalents regeneration mechanism (NADH and NADPH) essential for maintaining the redox balance (Remize et al., 2000). Enzymes involved in the PDH bypass include pyruvate decarboxylase (Pdc), acetaldehyde dehydrogenase (Ald), and acetyl-CoA synthetase (Acs) (**Figure 1**). The PDH complex leads to the formation of acetyl-CoA in the mitochondria through the oxidative decarboxylation of pyruvate. However, *S. cerevisiae* is unable to transport acetyl-CoA out of the mitochondria. Moreover, cytosolic NADP^+^-dependent Ald is active during alcoholic fermentation, while PDH activity is limited under anaerobic conditions (Remize et al., 2000). Therefore, the PDH bypass is necessary for providing acetyl-CoA in the cytosolic compartment which is used, *inter alia*, in lipid synthesis (for a review, see Pronk et al., 1996).

Pdc catalyzes the decarboxylation of pyruvate to acetaldehyde and carbon dioxide. In *S. cerevisiae*, Pdc is encoded by three structural genes, *PDC1*, *PDC5*, and *PDC6* which encode Pdc1, Pdc5, and Pdc6 isoforms, respectively (Hohmann, 1991; Pronk et al., 1996). Pdc1 and Pdc5 are 88% identical (Hohmann and Cederberg, 1990). Pdc1 is the predominant isoenzyme form, performing 80–90% of the activity in cells. The Pdc6p is an active Pdc (Hohmann, 1991; Zeng et al., 1993; Baburina et al., 1994) but is not apparently involved in glucose fermentation and its role remains unclear (Hohmann, 1991). The regulatory genes *PDC2*, *PDC3*, and *PCD4* encode probably positive transcriptional regulators required for high-level expression of structural *PDC1* and *PDC5* genes (Milanovic et al., 2012).

Ald is responsible for the conversion of acetaldehyde to acetate. The *S. cerevisiae* Ald family counts five isoenzymes localized in the mitochondria or the cytosol. Ald6 and Ald4 have been shown to be the main cytosolic and mitochondrial Ald, respectively. Cytosolic Ald is encoded by *ALD2*, *ALD3*, and *ALD6* (occasionally named *ALD1*) genes and the mitochondrial enzymes are encoded by *ALD4* (occasionally named *ALD7*) and *ALD5* genes (Navarro-Aviño et al., 1999). Ald6 uses the NADP^+^ co-enzyme, activated by Mg^2+^, and is not glucose-repressed (Dickinson, 1996; Meaden et al., 1997). Ald4 uses both the NAD^+^ and NADP^+^ co-enzymes activated by K^+^ and thiols, and it is highly glucose-repressed (Jacobson and Bernofsky, 1974). Numerous studies stated that cytosolic Ald is responsible for the formation of acetate from glucose and that mitochondrial enzymes are involved during growth on ethanol or glycerol as carbon sources (Saigal et al., 1991; Wang et al., 1998). Remize et al. (2000) showed that a strain deleted in the *ALD6* gene led to a considerable decrease in acetate yield. The absence of Ald6p was compensated by mitochondrial isoforms, involving the transcriptional activation of the *ALD4* gene (Saint-Prix et al., 2004). More recently, it was demonstrated that the fermentation stress response gene *AAF1* regulates acid acetic production under standard laboratory conditions. This gene encodes a probable transcription factor, containing a C2-H2 zinc finger domain at the N-terminus. Indeed, *AAF1* regulates the expression of *ALD4* and *ALD6* (Walkey et al., 2012). The deletion of this gene significantly reduced acetic acid levels without increasing the acetaldehyde concentration in wine (Luo et al., 2013).

Acs catalyzes the formation of acetyl-CoA from acetate. *S. cerevisiae* contains two structural genes *ACS1* and *ACS2*, each encoding an active Acs (Van den Berg et al., 1996). It has been shown that Acs is an essential enzyme in *S. cerevisiae*. A disruption of both *ACS1* and *ACS2* genes is lethal (Van den Berg and Steensma, 1995).

An imbalance of reduction equivalents at the beginning of *S. cerevisiae* growth in must, due to the initial lack of alcohol dehydrogenase, triggers another mechanism: glycerol production (Gancedo and Serrano, 1989) (**Figure 1**). Dihydroxyacetone phosphate, the substrate for the glycerol formation pathway, can be provided either by the glycolytic degradation of sugar or by gluconeogenic flux when non-fermentable carbon sources are used (Nevoigt and Stahl, 1997). Dihydroxyacetone phosphate is converted to glycerol-3-phosphate, which is an intermediate for glycerol formation. Two homologous genes *GPD1* and *GPD2* encode the isoenzymes glycerol-3-phosphate dehydrogenase (Gpd). *GPD1* expression is induced by osmotic stress. The repressor/activator Rap1p was demonstrated to be an important determinant of induced transcriptional activities of the *GPD1* promoter (Eriksson et al., 2000). Expression of *GPD2* is not affected by changes in external osmolarity, but it is stimulated by anoxic conditions (Ansell et al., 1997). A recent study by Pérez-Torrado et al. (2016) showed the induction of *GPD1* after the first hour of growth in wine fermentation conditions for different *Saccharomyces* species. For the *GPD2* gene, the time and the level of induction seem to be species- or strain-dependent. Moreover, some strains do not seem to activate this gene which presents very low mRNA levels.

In the present study, we performed sequential fermentations, combining *M. pulcherrima* and *S. cerevisiae* strains, in order to evaluate the effect of the presence of *M. pulcherrima* on the production of acetic acid and glycerol during alcoholic fermentation. Moreover, the impact of this sequential culture on the expression of genes in *S. cerevisiae* encoding enzymes involved in acetic acid and glycerol pathways during alcoholic fermentation was investigated.

## Materials and Methods

### Yeast Strains

The commercial strain *S. cerevisiae* PB2023 (SPINDAL-AEB group) was used as control strain. The non-*Saccharomyces M. pulcherrima* MCR-24 strain (accession number: JX234570) used in this study was previously isolated from Pinot Noir grape must. This strain was selected for its alcoholic fermentation performance (completion of alcoholic fermentation producing around 11% v/v ethanol) and its low acetic acid production (Sadoudi et al., 2012).

### Media

Sauvignon Blanc grape must (112 g l^-1^ glucose, 109 g l^-1^ fructose, 3.1 g l^-1^ L-malic acid, 378 mg l^-1^ total nitrogen, pH 3.35) supplemented with sulfur dioxide (30 mg l^-1^) was used in the fermentation tests. The must was pasteurized at 100°C for 10 min and the effectiveness of this treatment was verified by plating on YPD solid medium (20 g l^-1^ glucose, 5 g l^-1^ yeast extract, 10 g l^-1^ peptone, 0.2 g l^-1^ chloramphenicol, agar 20 g l^-1^). YPD liquid medium was used for yeast pre-cultures before inoculation in musts.

YPD solid medium was used for viable cell counting (non-*Saccharomyces* or *S. cerevisiae* yeasts) during mono-culture fermentations and total viable cell counting (both non-*Saccharomyces* and *S. cerevisiae* yeasts) during sequential fermentations.

Lysine agar (LA) medium [66 g l^-1^ Lysine medium (Oxoid), 10 ml 50% potassium lactate, 0.11 ml 90% lactic acid, and 0.2 g l^-1^ chloramphenicol] was used for viable cell counting of non-*Saccharomyces* yeast during sequential fermentation. LA medium is a selective medium which limits the growth of *S. cerevisiae* (Lin, 1975). The number of *S. cerevisiae* cells was given as the difference between the total plate count using YPD agar and the plate count using LA.

### Fermentation Conditions and Sampling

Fermentations were carried out for *S. cerevisiae* PB2023 in pure culture and *M. pulcherrima* MCR-24/*S. cerevisiae* PB2023 in mixed cultures.

#### Pure Cultures

Pure cultures were carried out in 500 ml Erlenmeyer flasks containing 350 ml of Sauvignon Blanc grape must and closed with dense cotton plugs. Yeasts were pre-cultured in YPD medium at 30°C for 48 h and then inoculated in musts at a concentration of 10^6^ cells ml^-1^. Fermentations were carried out in triplicate at 20°C, without shaking. Fermentation progress and yeast growth were monitored throughout the fermentation process by measuring sugar concentration and by viable cells counts.

#### Sequential Cultures

Sequential fermentations were carried out in 500 ml Erlenmeyer flasks containing 350 ml of the same must as described above. Before must inoculation, *S. cerevisiae* PB2023 and *M. pulcherrima* MCR-24 were pre-cultured in YPD medium for 48h. *M. pulcherrima* MCR 24 and *S. cerevisiae* PB2023 were then sequentially inoculated at a ratio of 10:1. *M. pulcherrima* MCR 24 was inoculated at 10^7^ cells ml^-1^ and after 48 h, *S. cerevisiae* PB2023 was introduced at 10^6^ cells ml^-1^. Each experiment was performed in triplicate at 20°C under static conditions. Fermentation progress and yeast growth were monitored throughout the fermentation process by measuring sugar concentration and by viable cell counts, as described previously.

#### Sampling

Samples of the fermenting must were taken at different stages of fermentation (-2, -1, 0, 1, 2, 3, 4, 5, 6, and 8 days of fermentation) from each fermentation trial. Day “-2” corresponds to the day of inoculation with *M. pulcherrima* MCR 24 strain and day “0” corresponds to the day when *S. cerevisiae* PB2023 was added. One part of each sample was used to determine the cell number. The other part of the sample was centrifuged at 1000 rpm for 5 min at 4°C. Supernatants were stored at -20°C and analyzed later to determine residual sugar, ethanol, glycerol, and acetic acid concentrations. The cell pellet was collected for RNA extraction. The RNA extractions were performed from the day “1” of fermentation until the end of the process.

### Enological Parameter Analysis

Glucose, fructose, ethanol, glycerol, and acetic acid were determined using enzymatic kits following the manufacturer’s instructions (Bio-SenTec, France). Total acidity was determined by the potentiometric method. The wine was decarbonated and then titrated by NaOH 0.1 N solution until pH 7. The result was expressed in g l^-1^ tartaric acid.

### RNA Extraction and Reverse Transcription (cDNA Synthesis)

Total RNAs extraction was performed using a commercial RNeasy kit (Qiagen) with slight modifications. After centrifugation, cells were added to the extraction buffer together with 600 μl of sterile glass beads (0.5 mm in diameter). The cells were then disrupted using the Precellys instrument (Bertin Technologies, France) at 6500 *g* for 30 s followed by chilling on ice for 30 s. This step was repeated six times. The extraction was then continued according to the manufacturer’s instructions (Qiagen).

The extracted RNA was quantified by measuring absorbance at 260 nm using a bio-photometer (Eppendorf). The RNAs (2 μg of total RNA) were treated with 5 U of DNase (Fermentas/Thermo Fisher Scientific, France) following the protocol described by the manufacturer. As a quality control assay, the absence of contaminant genomic DNA in RNA preparations was checked before cDNA synthesis using RNA as a template in real-time PCR assays (RNA not reverse-transcribed to cDNA). cDNA was then synthesized from 1 μg of total RNA in 20 μl reaction mixture using the iScript cDNA synthesis kit (Bio-Rad, France). Each RNA extraction was performed in triplicate.

### Primer Design

The primers for RT-PCR (target and housekeeping reference genes) given in **Table 1** were designed using the free online Primer3 0.4.0 software^1^. The primers were designed to have length about 18–22 bp, a G/C content of over 50%, and a Tm of about 60°C. The PCR product sizes ranged from 90 to 120 bp. Secondary structures and dimers formation were controlled with the Oligo Analyzer 1.0.3.0 software. Primer specificity and PCR product size were obtained *in silico* from the entire genome of the S288C strain^2^.

**Table 1:** Genes and primers used in RT-qPCR.

Genes	NCBI Gene ID^a^	Description	Forward and reverse primers 5′ → 3′	Primer size	PCR product salt (bp)^b^
*PDC1* (YLR044C)	850733	Pyruvate decarboxylase, isozyme 1	CTTACGCCGCTGATGGTTA	19	95
			GGCAATACCGTTCAAAGCAG	20	
*PDC5* (YLR134W)	850825	Pyruvate decarboxylase, isozyme 5	GGCTGATGCTTGTGCTTCTA	20	120
			GGGTGTTGTTCGTCAATAGC	20	
*ALD6* (YPL061W)	856044	Cytosolic aldehyde dehydrogenase, isozyme 6	TCTCTTCTGCCACCACTGAA	20	100
			CCTCTTTCTCTTGGGTCTTGG	21	
*ALD4* (YOR374W)	854556	Mitochondrial aldehyde dehydrogenase, isozyme 4	CGGGTTTGGTAAGATTGTGG	20	106
			TGCGGACTGGTAAATGTGTC	20	
*ACS2* (YLR153C)	850846	Acetyl-CoA synthase, isozyme 2	ATTGGTCCTTTCGCCTCAC	19	118
			GCTGTTCGGCTTCGTTAGA	19	
*ADH1* (YOL086C)	854068	Alcohol dehydrogenase, isozyme 1	GGTCACTGGGTTGCTATCTCC	21	107
			CCTTCACCACCGTCAATACC	20	
*ADH2* (YMR303C)	855349	Alcohol dehydrogenase, isozyme 2	TGCCCACGGTATCATCAAT	19	98
			GCAAACCAACCAAGACAACAG	21	
*CAT2* (YML042W)	854965	Carnitine acetyltransferase 2	CAAACTGATGACCCATGACG	20	94
			GGACTGCGATCCTTGGAATA	20	
*GPD1* (YDL022W)	851539	Glycerol-3-phosphate dehydrogenase isozyme 1	TTTTGCCCCGTATCTGTAGC	20	100
			TGGACACCTTTAGCACCAACT	21	
*PGK1* (YCR012W)	850370	3-Phosphoglycerate kinase, key enzyme in glycolysis and gluconeogenesis	GGTAACACCGTCATCATTGG	20	100
			AAGCACCACCACCAGTAGAGA	21	
*TDH2* (YJR009C)	853465	Glyceraldehyde-3-phosphate dehydrogenase, isozyme 2	AACATCATCCCATCCTCTACCG	22	94
			GGACTCTGAAAGCCATACCG	20	


*^a^Identification number; ^b^bases pairs.*

*PGK1* and *TDH2* genes (**Table 1**) were used as housekeeping reference genes because they were shown to be two genes whose expression remained stable and independent of growth conditions, as highlighted by (Vaudano et al., 2011).

Primers were purchased from Eurogentec, Belgium. In order to confirm the specificity of the primers only for *S. cerevisiae* genomic DNA in sequential culture samples, each couple of primers was tested in RT-qPCR using the genomic DNA of *S. cerevisiae* or *M. pulcherrima* as a template. No amplification was detected in the *M. pulcherrima* genomic DNA template (data not shown).

### Quantitative Real-Time PCR

Real time PCR was performed in 96-well plates on a CFX-96^TM^ Real Time system (Bio-Rad) using SYBR Green as fluorophore. Reactions were carried out in 25 μl of mix containing 12.5 μl of PCR master mix (Promega), 2.0 μl of primer mix (7 pM final concentration), 5.5 μl of DNase and RNase free H_2_O, and 5 μl of cDNA. Positive (*S. cerevisiae* genomic DNA as template) and negative (water as template) controls were also incorporated in each assay. The thermocycling program consisted of one hold at 95°C for 3 min; 40 cycles of 10 s at 95°C, 30 s at 60°C and 30 s at 72°C and a final extension at 72°C for 5 min. After the completion of the thermocycling program, melting curve data were then collected to verify PCR specificity, contamination and the absence of primer dimers. The melting curve was obtained by increasing the temperature from 60 to 95°C at 0.5°C/10 s.

The PCR efficiency of each primer pair (*E*) was evaluated by running a standard curve with serial dilution of cDNA. When *E* = 100%, the amount of PCR product can double in each cycle. Efficiencies and threshold cycle (*C*_T_) values were obtained by using the automated system software setting. The threshold cycle value was defined as the number of cycles required to reach a point in which the first fluorescent signal is recorded as statistically significant above background. In this study, the threshold fluorescence baseline was set manually at 100 relative fluorescence units (RFU).

The relative expression of a given gene was calculated using the 2^-ΔΔCT^ method (Livak and Schmittgen, 2001). The gene expression levels were given as a differential of the expression levels in *S. cerevisiae* in mixed culture conditions *versus* expression levels of *S. cerevisiae* in pure culture. The results were normalized by using two reference genes *PGK1* and *TDH2* (**Table 1**). The data were analyzed using the comparative critical threshold (ΔΔ*C*_T_) in which the amount of sample target RNA was adjusted to a control target RNA, where:

- Control: target RNA of *S. cerevisiae* from pure culture conditions
- Sample: target RNA of *S. cerevisiae* from mixed culture conditions

Δ*C*_T_ = *C*_T_ gene of interest -*C*_T_ reference gene

ΔΔ*C*_T_ = Δ*C*_T_ of sample - Δ*C*_T_ of control

Relative expression level = 2^-ΔΔC_T_^

We considered that genes were significantly down- or over-expressed if their relative expression level was found to be at least twofolds lower or higher than the control conditions as previously described (Desroche et al., 2005).

### Statistical Analysis

Metabolite concentrations were subjected to one-way analysis of variance (ANOVA) followed by a Tukey’s (HSD) *post hoc* test (confidence interval 95%) to test for significance differences between the wines.

## Results

### Fermentation Behavior of Pure and Sequential Cultures

**FIGURE 2:**
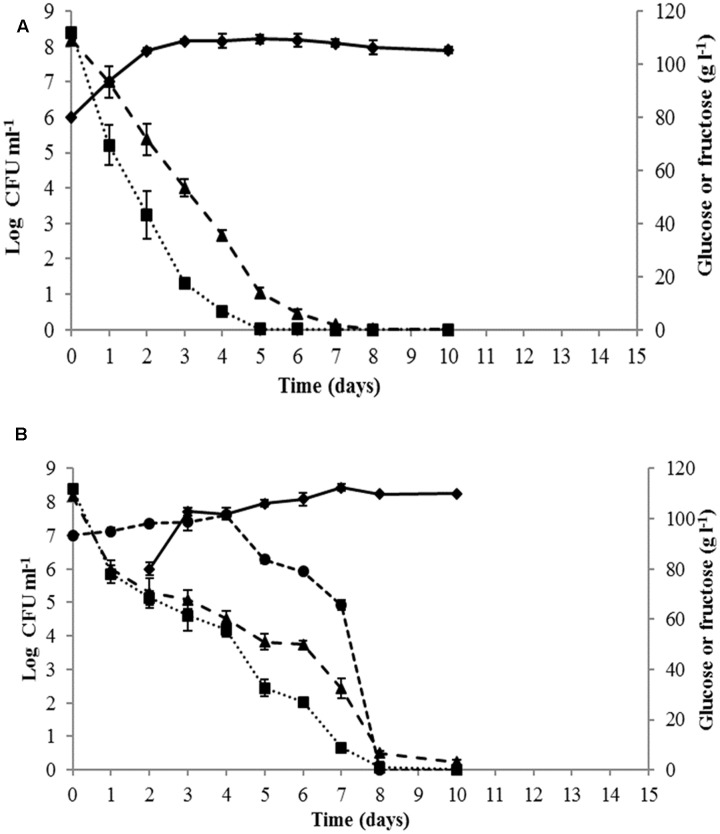
Growth kinetics and sugar consumption during mono-culture and sequential culture fermentations: **(A)** *S. cerevisiae* PB2023 (–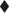
–), and **(B)** *M. pulcherrima* MCR 24 (–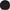
–)/*S. cerevisiae* PB2023 (–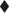
–). Glucose (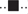) and fructose (–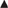
–). Data are representative of three independent trials.

Yeast growth dynamics and sugar consumption during must fermentation were monitored for single and sequential cultures (**Figure 2**). The fermentation kinetics of the control *S. cerevisiae* PB2023 pure culture indicated that the maximal population was reached after 3 days (1.4 × 10^8^ viable cells ml^-1^). This cell concentration was maintained until the end of fermentation (**Figure 2A**). *S. cerevisiae* completed the alcoholic fermentation in 8 days without remaining sugar. When the alcoholic fermentation was conducted with sequential culture of *M. pulcherrima* MCR-24 and *S. cerevisiae* PB2023 (inoculation ratio 10:1), the fermentation progressed to completion in 10 days (**Figure 2B**). The maximum population reached for *S. cerevisiae* was 3 × 10^8^ viable cells ml^-1^ and 4 × 10^8^ viable cells ml^-1^ for *M. pulcherrima*. The presence of *M. pulcherrima* did not affect the growth of the *S. cerevisiae* PB2023 strain. However, *M. pulcherrima* MCR 24 population dropped after the inoculation of *S. cerevisiae* PB2023 and no viable cells were detected after 8 days.

**FIGURE 3:**
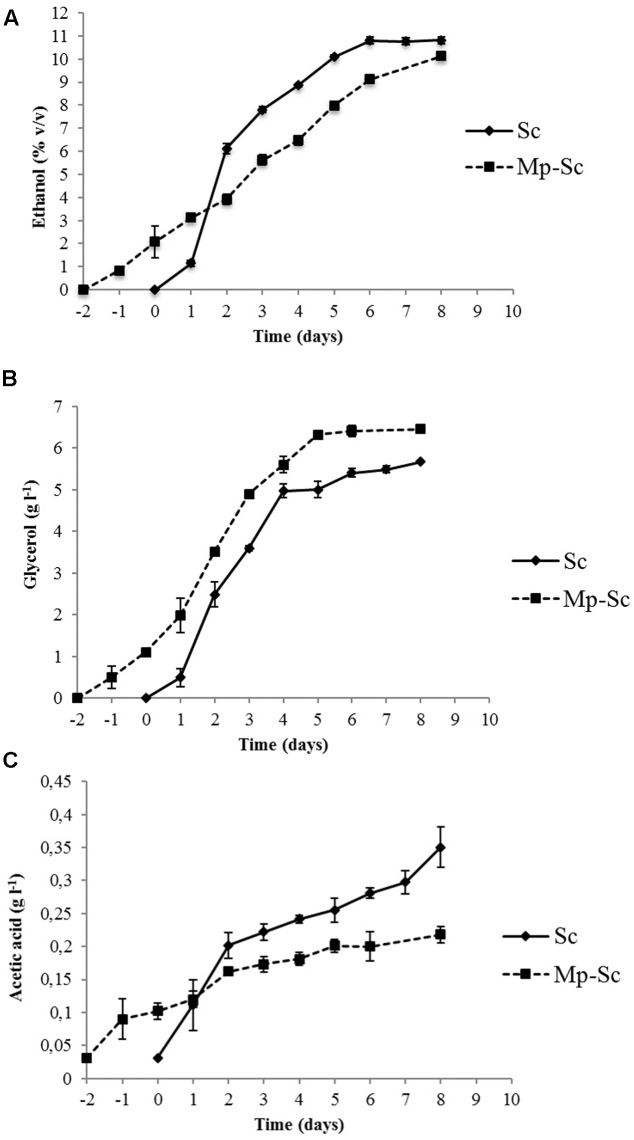
Evolution of metabolites production during alcoholic fermentation carried out by *S. cerevisiae* PB2023 (Sc) and *M. pulcherrima* MCR 24/*S. cerevisiae* PB2023 (Mp-Sc). **(A)** Ethanol production; **(B)** glycerol production; **(C)** acetic acid production. Day 0 corresponds to the day of inoculation with *S. cerevisiae* PB2023. Data are representative of three independent trials.

The evolution of ethanol showed different kinetics in sequential and pure fermentations (**Figure 3A**). During the first 72 h of fermentation, as expected, the *S. cerevisiae* pure culture produced ethanol faster and in higher concentration than that produced by sequential culture, after which production was progressive and at a lower rate until the end of fermentation (10.58% v/v). *M. pulcherrima*/*S. cerevisiae* sequential culture showed a lower but regular trend for ethanol production until the end of fermentation (10.14% v/v). In both cases, the fermentation yields were slightly higher than usual [21 g l^-1^ sugars for 1% (v/v) ethanol instead 16.8 g l^-1^]. These data were probably linked to winemaking trials in small volumes (350 ml).

*Saccharomyces cerevisiae* pure culture produced a higher amount of glycerol (4.97 g l^-1^) in the first 4 days of fermentation compared to the sequential culture (3.52 g l^-1^). After day 4, glycerol was produced gradually until the end of fermentation (5.67 g l^-1^). Sequential culture exhibited lower concentrations of glycerol in the first 4 days of fermentation, but its concentration was higher at the end of the process (6.46 g l^-1^) (**Figure 3B**).

The acetic acid production kinetics of pure and sequential cultures are shown in **Figure 3C**. Pure culture of *S. cerevisiae* produced significantly higher amounts of acetic acid (0.35 ± 0.01 g l^-1^) compared to sequential culture (0.21 ± 0.03 g l^-1^). For *S. cerevisiae* pure culture, 57% of the final amount was produced during the first 3 days of fermentation. Interestingly, the presence of *M. pulcherrima* in culture together with *S. cerevisiae* led to a reduction of acetic acid production from the beginning of fermentation.

### Gene Expression during Alcoholic Fermentations

**FIGURE 4:**
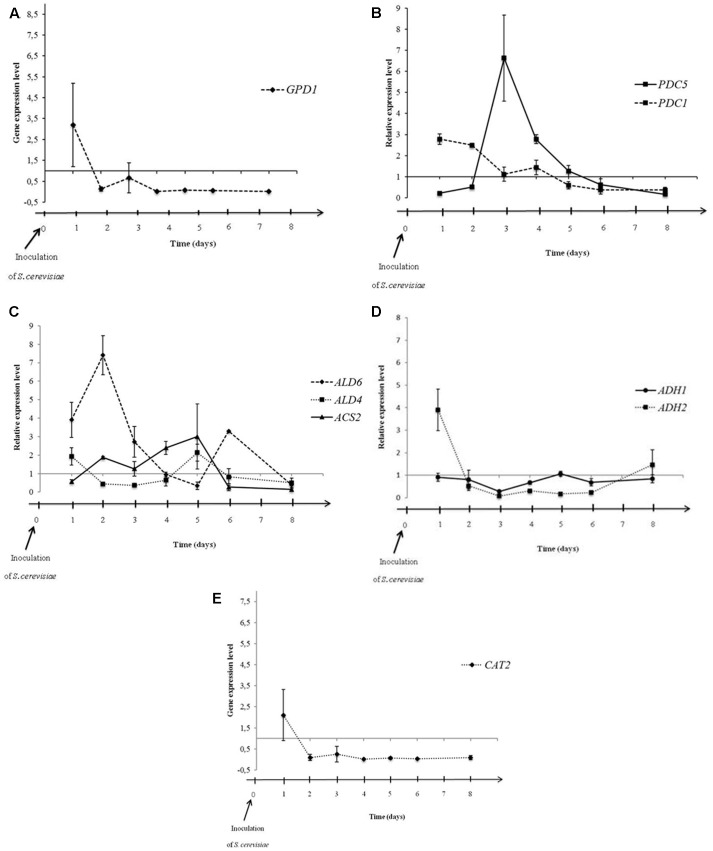
Expression levels of *GPD1* **(A)**, *PDC1* and *PDC5* **(B)**, *ALD6*, *ALD4*, and *ACS2* **(C)**, *ADH1* and *ADH2* **(D)**, and *CAT2* **(E)** in *S. cerevisiae* PB2023 at different stages of sequential culture fermentation. Data are representative of three independent trials. The gene expression levels were given as a differential of the expression levels in *S. cerevisiae* in sequential culture conditions *versus* expression levels of *S. cerevisiae* in pure culture.

Previous data suggested that the metabolic pathways could be affected by interactions occurring between both yeasts during alcoholic fermentation. In this context, we studied the influence of *M. pulcherrima* MCR 24 growth on acetic acid and glycerol productions of *S. cerevisiae* evaluating Pdc, aldehyde dehydrogenase, Acs, and alcohol dehydrogenase gene expression during alcoholic fermentations. These enzymes are the key enzymes involved in the acetic acid production pathway. We have added the analysis of the expression of Gpd. Gene expression in *S. cerevisiae* was evaluated in sequential culture relative to the gene expression of *S. cerevisiae* in pure culture (control) (**Figure 4**). Time 0 corresponds to the day of inoculation of the *S. cerevisiae* PB2023 strain in the sequential culture.

**Figure 4A** shows the differential gene expression level of *GPD1* in sequential culture condition. Dihydroxyacetone phosphate is converted to glycerol-3-phosphate, an intermediate for glycerol formation, by a Gpd enzyme encoded by the gene *GPD1* (**Figure 1**). The *GPD1* gene was over-expressed at 24 h after inoculation of *S. cerevisiae*, then the transcriptional level dropped and remained stable until the end of fermentation. This observation can be linked to the increase in the quantity of glycerol at the first 24 h of fermentation and then a similar production rate should be observed for *S. cerevisiae* in both fermentation conditions (pure and sequential culture) but it is hazardous to correlate this hypothesis with the analytical data shown **Figure 3B**. Indeed, *M. pulcherrima* MCR 24 produced glycerol (approximately 1 g l^-1^) before inoculation with *S. cerevisiae* and the levels measured after 48 h of fermentation may have resulted from the co-production of glycerol by both yeasts.

The differential of *PDC1* and *PDC5* gene expression levels during fermentation is shown in **Figure 4B**. The *PDC1* gene was slightly over-expressed at 24 and 48 h after inoculation. After that, gene expression decreased gradually until the end of fermentation. However, *PDC5* gene expression was not significantly affected by the sequential culture in the first 48 h but it was highly over-expressed at the 3rd day of fermentation (6.6-fold). Then, expression decreased gradually until the end of fermentation. Interestingly, we assume that Pdc encoding by both genes was not induced at the same time but alternately, confirming the hypothesis of their auto-regulation during alcoholic fermentation (Hohmann and Cederberg, 1990; Eberhardt et al., 1999). The alternate over-expression of the *PDC1* and *PDC5* genes was observed in the first 4 days of fermentation. After that, the transcriptional levels of both genes in sequential culture condition were identical to transcriptional levels of these genes in pure culture conditions. Furthermore, over-expression of these genes suggests that the sequential culture led to an increase in the production of acetaldehyde from pyruvate.

The differential expressions of genes directly involved in acetate production, i.e., *ALD6*, *ALD4*, *ACS2*, are presented in **Figure 4C**. The *ALD6* gene was over-expressed in the first 3 days of fermentation, reaching its maximum level of expression on the 2nd day (7.4-fold; **Figure 4B**). However, the mitochondrial *ALD4* gene was not over-expressed and remained stable during fermentation. This means that mitochondrial aldehyde dehydrogenase was not affected by the mixed culture condition, but cytosolic Ald6 activity could be privileged in order to regenerate the reduced co-enzyme NADPH (**Figure 1**). The *ACS2* gene encoding Acs did not present over-expression in the mixed culture condition. The *CAT2* gene encoding carnitine acetyltransferase was twofold lower expressed in sequential culture condition (**Figure 4E**).

The expression levels of genes *ADH1* and *ADH2* encoding alcohol dehydrogenase are shown in **Figure 4D**. No over-expression was observed in the *ADH1* gene during fermentation. In contrast, the *ADH2* gene was highly over-expressed 24 h after inoculation of *S. cerevisiae* (fourfold), which is involved in the conversion of ethanol into acetaldehyde (**Figure 1**). After 24 h, the *ADH2* gene expression level dropped rapidly and a down regulation of *ADH2* was observed from the 3rd to the 6th day of fermentation.

## Discussion

The early inoculation of *M. pulcherrima* MCR 24 did not compromise the growth of *S. cerevisiae* PB2023, preventing the risk of a sluggish or a stuck alcoholic fermentation. Moreover, the *M. pulcherrima* population dropped after the inoculation of *S. cerevisiae* and no viable cells were detected after 8 days (**Figure 2B**). Such an antagonistic effect has been reported previously (Jolly et al., 2003; Rodríguez et al., 2010; Comitini et al., 2011; Sadoudi et al., 2012). This result could not be linked to intolerance to ethanol concentration, since we previously demonstrated that the MCR 24 strain can produce approximately 10% v/v ethanol (Sadoudi et al., 2012). According to Nguyen and Panon (1998), the antagonistic effect could be attributed to killer toxins. Another explanation is the interaction occurring between both yeasts, mediated by the cell–cell contact mechanism (Nissen and Arneborg, 2003) or competition between yeasts for the nutrients available in the must. *S. cerevisiae* PB2023 grew faster than *M. pulcherrima* MCR 24 and thus it could impoverish the medium. Sequential inoculation did not affect the ethanol level in the wine despite the death of *M. pulcherrima*. On the other hand, it induced a significant increase in glycerol content and a decrease in acetic acid concentration (**Figure 3**). These data confirm the benefits of using *M. pulcherrima* prior the inoculation of the *S. cerevisiae* starter, in accordance with previous results (Bely et al., 2008; Comitini et al., 2011), but they do not explain the positive impact of *M. pulcherrima* on *S. cerevisiae* metabolism.

All previous analytical data suggest that the metabolic pathways could be rerouted by interactions occurring between both yeasts during alcoholic fermentation. During the latter, acetic acid is produced *via* the cytosolic PDH bypass. In aerobic conditions, the PDH complex leads to the formation of acetyl-CoA in the mitochondria by oxidative decarboxylation of pyruvate. However, in fermentative conditions, the conversion of pyruvate to acetyl-CoA can occur *via* an indirect route, involving Pdc (which is also a key enzyme in alcoholic fermentation), Ald and Acs. This bypass route is the source in the cytosolic compartment of acetyl-CoA, which is used for lipid synthesis and acetate which can be precursor of volatile esters.

The production of glycerol involves the reduction of dihydroxyacetone phosphate derived from the glycolytic degradation of sugar. The NAD^+^-dependent Gpd catalyzes the first step in glycerol production. This metabolism also permits the regeneration of reducing equivalents (NADH), more particularly at the beginning of *S. cerevisiae* growth in fermentative conditions.

The over-expression of *PDC1* and *PDC5* encoding two isoforms of Pdc and the *ALD6* gene encoding cytosolic aldehyde dehydrogenase (**Figures 4B,C**) leads to the assumption of an over production of acetic acid by-product, which appears inconsistent with the analytical data which shows that acetate was reduced in mixed culture condition. One explanation could be due to the conversion of acetate into acetyl-CoA used in other metabolic pathways such as lipid synthesis or esterification related to the production of esters. Indeed, we previously observed higher levels of acetate esters in Sauvignon wine from a *M. pulcherrima*/*S. cerevisiae* sequential culture (Sadoudi et al., 2012). However, it is clear that acetyl-CoA was not transported into mitochondria since the *CAT2* gene encoding carnitine acetyltransferase was under-expressed in sequential culture condition (**Figure 4E**).

The lower acetate production could not be due ethanol production since the ethanol contents are comparable under the two fermentation conditions. Another hypothesis that could explain our analytical data is that a part of dihydroxyacetone phosphate is used for glycerol production at the beginning of fermentation. Glycerol can be produced mostly at the beginning of fermentation in response to hyper osmotic conditions (high concentration in sugars). Moreover, anaerobic conditions require the production of endogenous electron acceptors and glycerol production can serve as a redox valve to eliminate excess reducing power in *S. cerevisiae* (Ansell et al., 1997). The *M. pulcherrima* strain MCR 24 may have depleted oxygen in the must during sequential culture, since it was inoculated 48 h before *S. cerevisiae*. Oxygen depletion (anaerobiotic conditions) could explain the modulation of glyceropyruvic fermentation and the orientation of metabolism to the PDH bypass, leading to the production of acetate and glycerol. These metabolism orientations are necessary to maintain the redox balance by regenerating NAD and NADH co-enzymes. Furthermore, increased glycerol formation requires an equimolar amount of cytoplasmic NADH. This requirement could be satisfied by a lower reduction of acetaldehyde to ethanol on the one hand and an increase in oxidation to acetate on the other (Blomberg and Adler, 1989; Nevoigt and Stahl, 1997). Therefore an increase in acetate production is usually accompanied by an increase in glycerol formation; however, a high levels of glycerol is not necessarily accompanied by high levels of acetic acid or acetaldehyde (Remize et al., 2000).

Independently of the expression of genes involved in acetate and glycerol production pathways, we hypothesized the possible consumption by *M. pulcherrima* MCR 24 of part of the acetate produced by *S. cerevisiae* in sequential culture fermentation. We performed a mono-culture with the *M. pulcherrima* MCR 24 strain using standardized grape juice supplemented with 1.5 g l^-1^ of acid acetic and observed the consumption of 0.57 g l^-1^ of the initial acetic acid during 8 days of fermentation (data not shown).

## Conclusion

This work is the first attempt to investigate *M. pulcherrima* and *S. cerevisiae* yeast–yeast metabolic interaction, reflected by gene expression in the acetic acid and glycerol production pathway in *S. cerevisiae* during controlled sequential fermentation in winemaking. The environmental changes in must induced by the presence of *M. pulcherrima* induced the alteration of the entire acetic acid and glycerol metabolic pathway of *S. cerevisiae.*

Future accession to the *M. pulcherrima* genome may provide very interesting investigative leads on the nature of interactions occurring in sequential fermentations at the transcriptomic level.

## Author Contributions

MS designed the experiments, analyzed the data, and wrote the manuscript. SR analyzed the data and wrote the manuscript. VD analyzed the data. HA and RT-M supervised the study.

## Conflict of Interest Statement

The authors declare that the research was conducted in the absence of any commercial or financial relationships that could be construed as a potential conflict of interest.

## Footnotes

1 http://frodo.wi.mit.edu/primer3/

2 http://www.ncbi.nlm.nih.gov/tools/primer-blast/index.cgi?LINK_LOC=BlastHome
